# Coding-Complete Genome Sequence of a *Falcon aviadenovirus A* Strain Associated with Necrotizing Hepatitis in an American Kestrel (*Falco sparverius*)

**DOI:** 10.1128/mra.00009-22

**Published:** 2022-03-17

**Authors:** Carl A. Gagnon, Chantale Provost, Stéphane Lair

**Affiliations:** a Swine and Poultry Infectious Diseases Research Center, Faculté de Médecine Vétérinaire, Université de Montréal, Saint-Hyacinthe, Québec, Canada; b Centre de Diagnostic Vétérinaire de l’Université de Montréal, Faculté de Médecine Vétérinaire, Université de Montréal, Saint-Hyacinthe, Québec, Canada; c Centre Québécois sur la Santé des Animaux Sauvages-Canadian Wildlife Health Cooperative, Faculté de Médecine Vétérinaire, Université de Montréal, Saint-Hyacinthe, Québec, Canada; DOE Joint Genome Institute

## Abstract

A necropsy was performed on an American kestrel (*Falco sparverius*) with necrotizing hepatitis associated with inclusion bodies, suggesting an adenovirus infection. A next-generation sequencing assay was conducted on the liver, and the coding-complete genome sequence of a *Falcon aviadenovirus A* strain was revealed.

## ANNOUNCEMENT

Adenoviruses possess a wide host spectrum, being found in numerous species of mammals, birds, and reptiles (1). The *Aviadenovirus* genus of the *Adenoviridae* family is entirely composed of viruses infecting different bird species, such as turkey, duck, goose, and chicken (1, 2). In chicken, aviadenoviruses are responsible for important diseases such as inclusion body hepatitis (3). The livers of other bird species, including falcons such as the northern aplomado falcon (*Falco femoralis*) and the American kestrel (*Falco sparverius*), are targeted by aviadenoviruses (4, 5). The strategy usually used to characterize adenoviruses, including the falcon adenovirus (FaAdV), is mainly through the sequencing of a portion of the hexon gene, which represents only around 2 to 4% of the viral genome. Aviadenoviruses possess the largest viral genomes of *Adenoviridae*, which are around 40,000 to 45,000 nucleotides (nt) of double-stranded DNA (2). As of now, the entire coding sequence of FaAdV (official viral species name, *Falcon aviadenovirus A*) is unknown. The main objectives of the present study were to confirm the adenovirus infection in the American kestrel and then to improve the FaAdV genome data.

**FIG 1:**
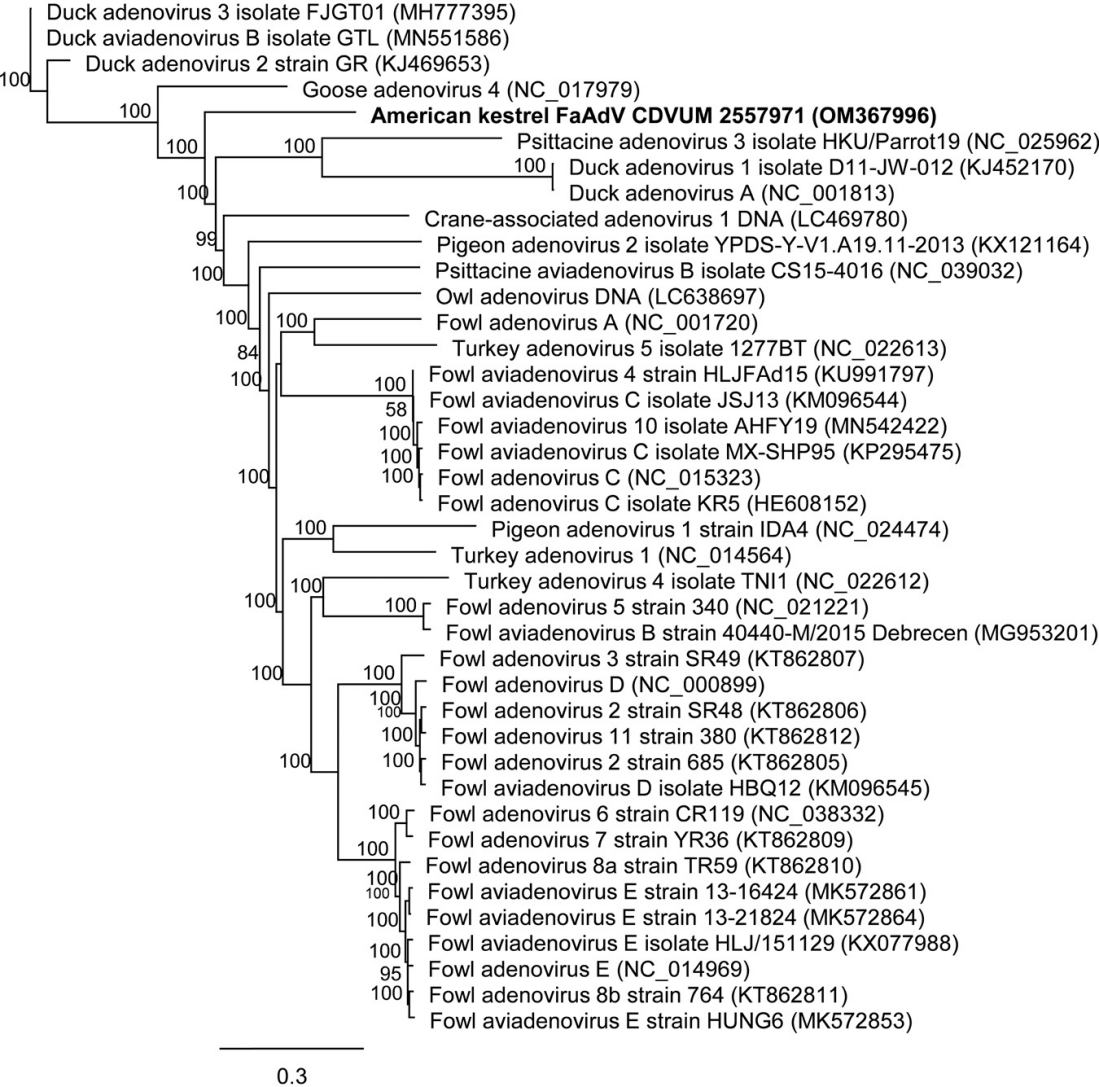
Phylogenetic tree of aviadenovirus complete genomes. Alignment was performed using MAFFT with default settings. The tree was generated with the Geneious Tree Builder application using the Jukes-Cantor and neighbor-joining building methods with 1,000 bootstraps. For all strains, GenBank accession numbers are indicated in parentheses.

A juvenile American kestrel was recovered from the wild (Papineauville, Québec, Canada [45.6192°N, 75.0189°W]) in August 2021. The bird, which was admitted to the Université de Montréal Raptor Rehabilitation Program for rehabilitation, died 19 days later. Death was attributed to Pasteurella multocida polyarthritis. However, acute multifocal necrotizing hepatitis with large basophilic intranuclear inclusion bodies was observed. An in-house quantitative PCR (qPCR) diagnostic assay specific for the detection of fowl adenoviruses was performed by our American Association of Veterinary Laboratory Diagnosticians (AAVLD)-accredited laboratory (Centre de Diagnostic Vétérinaire de l’Université de Montréal) (protocol PON-MOL-0094). A negative result was obtained. The supernatant of the liver tissue lysate was centrifuged for 5 minutes at 5,000 g; the supernatant was filtered using a 0.2-μm Amicon filter for 15 minutes at full speed and then was filtered again using an Amicon filter with a 50,000 nominal molecular weight limit (NMWL) during 30 minutes at 14,000 g (MilliporeSigma, Oakville, Ontario, Canada), from which the concentrated fraction on top of the filter was conserved. Total DNA was extracted from that fraction using the Quick-DNA/RNA kit (Zymo Research, Irvine, CA, USA). DNA was quantified using a high-sensitivity (HS) DNA kit with the Qubit apparatus (Thermo Fisher Scientific, Waltham, MA, USA). A sequencing library was generated using the Nextera XT kit and was sequenced with a MiSeq system with a 600-cycle v3 cartridge (Illumina, San Diego, CA, USA). The FaAdV genome sequence was obtained after trimming of adaptors and reads with quality scores of <0.05 using the *de novo* metagenome application with a minimum contig length at 1,000 nt and the scaffolding option selected in CLC Genomic Workbench software v21.0.5 (Qiagen, Hilden, Germany). A total of 239 contigs were obtained, and most of them were host-related sequences. All sequences were identified with the Standard Nucleotide BLAST online tool (NCBI, Bethesda, MD, USA). A total of 2,537,246 reads were obtained, including 37,995 FaAdV-specific reads, which resulted in a viral genome sequence length of 39,008 nt, with an average coverage of 125× and a GC content of 44,1%. Coding sequences were determined using predict genes in GLIMMER and transfer annotation in Geneious Prime v2022.0.1 (Biomatters Ltd., Shortland, New Zealand) with the closest full-length genome sequence found in GenBank, i.e., *Fowl aviadenovirus E* strain HUNG6 (GenBank accession number MK572853), which shares 72.8% nucleotide identity for 3% of its sequence with the American kestrel FaAdV genome using the BLAST online tool. The two coding-complete genomes share only 46.95% identity using MAFTT alignment in Geneious Prime. The longest and closest available reference nucleotide sequence (6,257 nt, with 98.9% nucleotide identity) found using the BLAST online tool was the northern aplomado FaAdV (GenBank accession number AY683541). The only other American kestrel FaAdV sequence reported was published in 2007 (5). That partial hexon gene sequence is 1,276 nt long and shares 99.9% nucleotide identity with our strain with MAFFT alignment. Overall, these findings demonstrate that our FaAdV strain is genetically far from any previously published aviadenovirus complete sequences (Fig. 1).

### Data availability.

The complete annotated coding sequence of the American kestrel FaAdV strain CDVUM 2557971 is available in GenBank (accession number OM367996), and the high-throughput sequencing data are available in the SRA database (accession number SRP360558).
